# Click Chemistry in Peptide-Based Drug Design

**DOI:** 10.3390/molecules18089797

**Published:** 2013-08-16

**Authors:** Huiyuan Li, Rachna Aneja, Irwin Chaiken

**Affiliations:** Department of Biochemistry and Molecular Biology, College of Medicine, Drexel University, 245 N 15th Street, New College Building, Room 11102, Philadelphia, PA 19102, USA; E-Mails: lindalihuiyuan@gmail.com (H.L.); Rachna.Arora@drexelmed.edu (R.A.)

**Keywords:** click chemistry, triazoles, application, peptides, drug discovery

## Abstract

Click chemistry is an efficient and chemoselective synthetic method for coupling molecular fragments under mild reaction conditions. Since the advent in 2001 of methods to improve stereochemical conservation, the click chemistry approach has been broadly used to construct diverse chemotypes in both chemical and biological fields. In this review, we discuss the application of click chemistry in peptide-based drug design. We highlight how triazoles formed by click reactions have been used for mimicking peptide and disulfide bonds, building secondary structural components of peptides, linking functional groups together, and bioconjugation. The progress made in this field opens the way for synthetic approaches to convert peptides with promising functional leads into structure-minimized and more stable forms.

## 1. Introduction

Peptide-based drugs are becoming an important component of the pharmaceutical drug market, especially since new techniques have been developed in recent years to improve production, reduce metabolic breakdown, and introduce alternative routes of administration. Peptides are generally considered to have low bioavailability and metabolic stability and therefore are not considered as good drug candidates. Nonetheless, compared with proteins and antibodies, peptides are less immunogenic and have the potential to penetrate into organs and tissues owing to their smaller size [1]. In addition, peptides have lower manufacturing costs and greater stability [1]. The application of modern synthetic techniques has dramatically accelerated the development of peptide drugs, such as solid-phase peptide synthesis [2] and native chemical ligation [3]. In 2001, a highly chemoselective and stereospecific Cu(I) catalyzed [3+2] cycloaddition reaction, often referred to as “click chemistry”, was conceived by Sharpless *et al.* and Meldal and colleagues [4,5] and has greatly enhanced access to chemical space of peptide-based components. Some other reactions, such as thio-ene click reaction and Diels-Alder reaction, are also considered as click chemistry. In this current review, we limit our discussion to the Cu(I) catalyzed [3+2] cycloaddition reaction. Click chemistry encompasses powerful, highly reliable, and selective reactions to generate substances by joining small azide and alkyne units together through heteroatom links in the presence of copper(I) catalysts. This chemistry has provided a rapid means to generate structural diversity in peptide scaffolds.

**Scheme 1:**
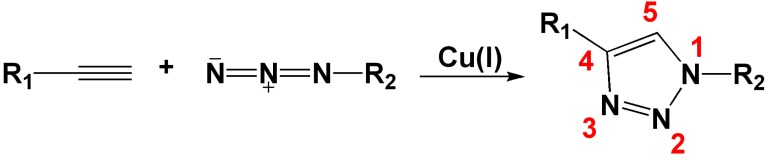
Cu(I) catalyzed Huisgen 1,3-dipolar cycloaddition reaction.

The click reaction is one type of Huisgen cycloaddition reaction [6,7], in which dipolarophiles react with 1,3-dipoles to form five-membered heterocycles including triazoles. Because the original Huisgen reaction was non-regioselective and required high temperature and pressure, it was largely ignored for decades. In 2002, Meldal and coworkers reported that the use of catalytic amounts of Cu(I) led to fast, highly efficient and regioselective azide–alkyne cycloadditions at room temperature in organic medium (Scheme 1) [5]. Shortly after, Sharpless and Fokin demonstrated that copper-catalyzed azide-alkyne cycloaddition (CuAAC) can be successfully performed in polar media such as *t*-butyl alcohol, ethanol or pure water [8]. These findings led to a remarkable escalation in use of Huisgen cycloadditions, and, within the last few years, CuAAC has been exponentially investigated in organic synthesis, inorganic chemistry, polymer chemistry and biochemistry [9,10,11,12,13]. Recently, new developments in the click chemistry field, such as ruthenium-catalyzed azide-alkyne cycloaddition [14] and copper-free click chemistry [15,16], have attracted much attention, especially in the application of copper-free click chemistry to biological systems [17,18]. Sharpless *et al.* [4] defined the rules of click reaction: A reaction must be modular, wide in scope, give very high yields, generate only inoffensive byproducts that are easily separated, and be stereospecific. The process must include simple reaction conditions, readily available starting materials and reagents, the use of no solvent, or a solvent that is benign or easily removed, and simple product isolation.

**Figure 1:**
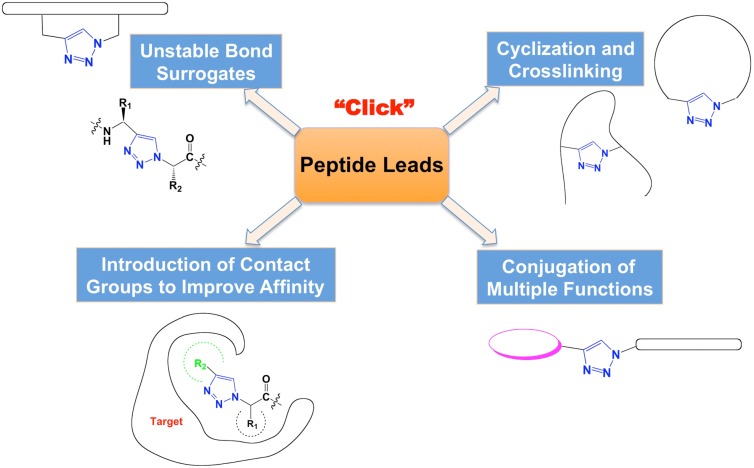
Types of click chemistry applications in peptide-based drug discovery.

The advantages of click reaction have been applied broadly in drug research in the last ten years [19,20,21,22]. In this review, we focus on the application of CuAAC click chemistry in peptide-based drug discovery (Figure 1).

## 2. 1,2,3-Triazoles as Surrogates for Unstable Bonds

The stability of natural peptides is one of the principal limitations of their use as drug candidates. Natural peptide bonds are subjected to proteolysis by various proteases. The disulfide bond usually stabilizes the secondary or tertiary structure in peptides and proteins. However, it is unstable in redox or thiol/disulfide exchange conditions. The replacement of unstable bonds with non-natural stable structures, while at the same time maintaining biological activity, can be useful for improving the drugability of peptides.

### 2.1. Bioisostere of Amide Bond

**Figure 2:**
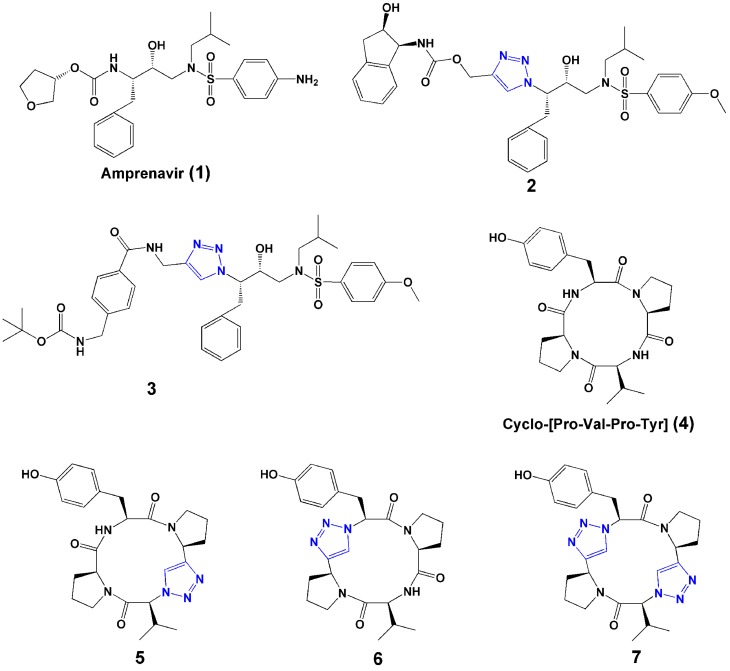
Selected examples of 1,2,3-triazoles used as surrogates of the amide bond.

Normally, peptides contain l-amino acids linked by amide bonds, which are susceptible to enzymatic peptide bond cleavage [1]. Proteases and peptidases, such as trypsin, α-chymotrypsin, and dipeptidyl-peptidase IV, are able to cleave specific as well as more generic sites in proteins and peptides [1]. Many strategies have been used to increase the stability of peptide drug candidates, such as introducing non-natural amino acids, terminal protection, cyclization, and backbone modification. Backbone modifications, such as NH-amide alkylation, replacement of the carbonyl function of the peptide bond by CH_2_ (amine, -CH_2_-NH-), and NH-amide bond exchange by O (ester, -CO-O-), S (thioester, -CO-S-), or CH_2_ (ketomethylene, -CO-CH_2_-), have all been used to increase plasma stability of the peptide [1]. The 1,2,3-triazole has attracted increasing attention as a bioisostere of the amide bond moiety of peptides. The physicochemical properties of 1,2,3-triazole have been reviewed [22]. The similarity of the two moieties can be seen in their sizes (distances between substituents are 3.8–3.9 Å in amides and 5.0–5.1 Å in 1,2,3-triazoles), dipole moment (amide ~ 4 Debye, 1,2,3-triazole ~ 5 Debye), and H-bond acceptor capacities [19,22]. The 1,2,3-triazole rings, with sp^2^-hybridized nitrogen atoms N(2) and N(3), can function as weak hydrogen-bond acceptors. And, the strong dipole moment of the 1,2,3-triazole ring polarizes the C(5) proton to such a degree that it can function as a hydrogen-bond donor similar to the amide NH. Furthermore, the 1,2,3-triazole ring has a large dipole that could align with that of the other amides in a given peptide secondary structure. Importantly, 1,2,3-triazoles are extremely stable to hydrolysis. Overall, 1,2,3-triazoles can be potential surrogates of amide bonds for peptide modification. Examples of this are shown in Figure 2.

Brik *et al.* [23] evaluated the copper(I)-catalyzed azide-alkyne [3+2] cycloaddition reaction for preparation of HIV-1 protease inhibitors. Over 100 compounds were synthesized in microtiter plates and screened *in situ*. Two compounds (**2** and **3**, Figure 2) showed the greatest activities against wild type and mutant HIV-1 proteases. Co-crystal structures with HIV-1 protease showed that inhibitors were bound in a position identical to that of parent compound amprenavir (**1**, Figure 2), arguing that the triazole was functioning as an excellent mimic of the peptide bond group in this class of molecules.

Bock et al. reported the synthesis of triazole-containing analogues (**5–7**, Figure 2) of the naturally occurring tyrosinase inhibitor cyclo-[Pro-Val-Pro-Tyr] (**4**, Figure 2) and showed that the analogues retained enzyme inhibitory activity, demonstrating the effectiveness of a 1,4-connected 1,2,3-triazole as a *trans* peptide bond isostere without compromising biological activity [24].

Horne *et al.* [25] found that triazoles can be used as amide bond surrogates in α-helical coiled coils. They selected the pLI mutant (see definition in [26]) of the α-helical coiled coil GCN4 as a model peptide in order to test the utility of the triazole substitution in the context of a peptide with well-defined secondary and quaternary structure in solution and in the solid state. The results showed that the modified peptides retained a native-like α-helical structure.

Horne *et al.* [27] designed and synthesized a series of cyclic pseudotetrapeptides containing 1,4 or 1,5-disubstituted 1,2,3-triazoles, which served as surrogates for *trans* or *cis* amide bonds, respectively. The 1,5-disubstituted 1,2,3-triazole was prepared by a thermal Huisgen [3+2] dipolar cycloaddition with DMF as solvent at 220 °C. To investigate the importance of configuration for binding affinity, they replaced an amide bond with either a 1,4 or 1,5-disubstituted 1,2,3-triazole. The heterocyclic compounds adopted conformations that corresponded closely to the targeted conformations of apicidin and demonstrated potent histone deacetylase (HDAC)-inhibitory activities, in some cases equivalent or superior to those of the natural product [27]. The study highlighted the utility of triazole-modified cyclic peptides in the construction of useful bioactive probe molecules, and supported the *cis-trans-trans-trans* conformation as the bioactive conformation of cyclic-tetrapeptide HDAC inhibitors.

### 2.2. Replacement of Disulfide Bond

In 2011, Empting *et al.* first used the 1,5-disubstituted 1,2,3-triazole as a surrogate of a disulfide bond [28]. The 1,5-disubstituted 1,2,3-triazole was generated in a ruthenium(II)-catalysis variation (RuAAC) of the CuAAC. They introduced 1,4- and 1,5-disubstituted 1,2,3-triazoles into a monocyclic variant of sunflower trypsin inhibitor-I (SFTI-1[1,14]) with the click reaction to replace a disulfide bridge, and showed that the 1,5-disubstituted triazole analogue retained nearly full biological activity in contrast to the 1,4- disubstituted triazole analogue. In the same year, Holland-Nell *et al.* [29] used 1,4-disubstituted 1,2,3-triazoles to replace two disulfide bonds in tachyplesin I (TP-I), a 17-residuce bicyclic peptide with antimicrobial activity. The TP-I has a β-hairpin ribbon structure in its active form; the triazole bridged analogue mimicked the secondary structure of wild-type TP-1 and released antimicrobial activity [29].

### 2.3. Substitution of Aromatic Rings and Double Bonds

In addition to being used as surrogates of amide and disulfide bonds, the 1,2,3-triazole also has been used to mimic aromatic rings [22], acyl-phosphates [19], and *trans*-olefinic moieties [19,22] in small molecule drug discovery. There are no reports as yet for such substitution in peptide drug modification. 1,2,3-triazoles are basic aromatic heterocylic compounds and might not be straightforward for replacement of neutral aromatic Phe and Tyr side chains. However, in some cases the replacement could help enhance binding affinity or bioactivity. Hence, it could be considered in future peptide drug discovery.

## 3. Crosslinking and Cyclization to Stabilize Functional Structures

Natural peptides tend to be conformationally quite flexible, which can limit the ability of a peptide drug candidate to achieve a specific and robust mode of action and hence present another important obstacle of peptides as drugs. Chemical modification, such as secondary structure mimicry or cyclization, could increase the structural rigidity and potentially restrict the structure into the bioactive conformation. 1,2,3-triazoles have been applied to mimic β-turn, *cis/trans* conformation, and α-helical structures.

### 3.1. β-turn Surrogate

**Figure 3:**
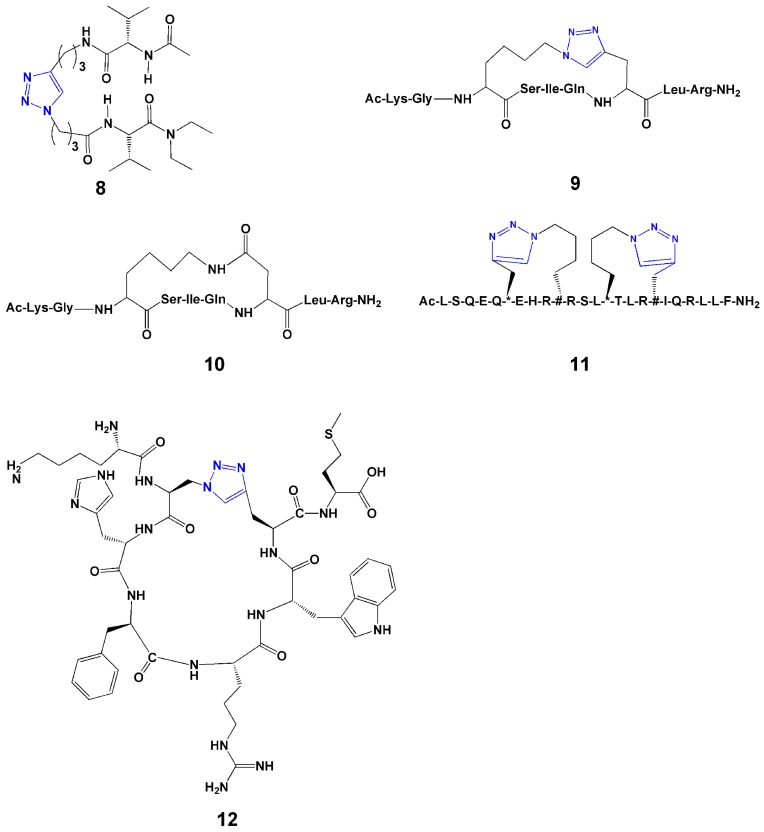
Selected examples of 1,2,3-triazoles used for secondary structure mimicry.

Guan and coworkers developed an efficient convergent strategy for constructing a β-turn mimicking unit through Cu(I)-catalyzed alkyne-azide cycloaddition between the termini of two peptide strands [30]. Their initial molecular modeling showed that a 1,4-connected 1,2,3-triazole ring might provide a geometry similar to that of a β-turn. This led to the proposal that cycloaddition between peptide strands derivatized with azides and terminal alkynes may provide a means to synthesize β-turn units. They designed and synthesized a series of 1,4-disubstituted 1,2,3-triazole-based tetrapeptides with various spacer lengths, and characterized these by NMR and FT-IR. They found that the tendency of β-turn formation for the triazole system strongly depends on the linker length. A three-carbon linker (**8**, Figure 3) is optimal for stable β-turn formation.

In 2009, Beierle *et al*. [31] designed and synthesized 13- and 14-membered-ring pseudotetrapeptides that contain either one or two triazole moieties with CuI-catalyzed alkyne-azide cycloaddition, respectively. All 16 compounds containing a 1,4-disubstituted 1,2,3-triazole adopted a distinct, rigid, conformationally homogeneous turn-like structure as demonstrated by NMR [31].

### 3.2. Cis/Trans Conformation

As mentioned earlier, Horne *et al.* designed 1,4 or 1,5-disubstituted 1,2,3-triazoles to mimic *trans* and *cis* amide bonds, respectively, as selective HDAC inhibitors [27]. Using 2D-NMR techniques and distance geometry calculations, they evaluated the structural differences between the 1,4- or 1,5-disubstituted 1,2,3-triazole analogues. The structural and functional properties suggested that subtle conformational differences would affect the HDAC inhibitory activities.

The ratio of *cis/trans* prolyl energy levels plays a crucial role in various biological processes. Paul et al. designed and synthesized four types of Pro-triazolopeptides (1,4-, 1,5-, 4,1-, and 5,1-disubstituted triazoles, counting from the N-terminal side) to investigate and fine-tune the *cis/trans* ratio of the dipeptide. The results demonstrated the coordinated adjustability of both the *cis*-percentage and the conformational stability toward intramolecular H-bonding effects [32].

### 3.3. Stabilizing α-Helical Structure

Intramolecular side-chain to side-chain cyclization of linear peptides using click reactions has been employed to achieve rigidification that results in either (1) restricting the conformational freedom toward a specific ensemble of bioactive conformations or (2) reducing susceptibility toward proteolytic enzymes, thus increasing metabolic stability *in vitro* and more significantly *in vivo*. Cantel *et al.* [33] explored synthetic routes of the cyclopeptide [Ac-Lys-Gly-Xaa(&^1^)-Ser-Ile-Gln-Yaa(&^2^)-Leu-Arg-NH_2_][&^1^(CH_2_)_4_-1,4-[1,2,3] triazolyl-CH_2_&^2^)] (**9**, Figure 3), a model i-to-i+4 side-chain to side-chain 1,4-disubstituted [1,2,3]triazolyl-bridged peptide derived from a modified fragment of N^α^Ac-hPTHrP-(11-19)NH_2_, and compared its solution conformation to the corresponding lactam analogue (**10**, Figure 3).

Comparison of the most representative NMR structures of lactam- and [1,2,3]triazolyl-containing cyclopeptide showed that both peptides assumed an α-helical structure in the cyclic part of the molecules. In follow-up work, Scrima *et al*. [34] synthesized the i-to-i+4 side-chain to side-chain cyclization, with variations in the size of the disubstituted 1,2,3-triazolyl-containing bridge. Structural studies showed that the 1,2,3-triazolyl flanked by a total of 5 or 6 -CH_2_- groups nicely accommodated α-helical structures and very closely reproduced the helical structure stabilized by a lactam bridge.

Jacobsen *et al.* [35] installed an i-to-i+3 constraint by a side-chain to side-chain CuAAC cycloaddition reaction in a model aminoisobutyric acid (Aib) rich peptide and examined the structural effects of side-chain to side-chain cyclization by NMR, X-ray diffraction, linear IR and femtosecond 2D IR spectroscopy. The data showed that the resulting cyclic peptide represented a more ideal 3_10_-helix than its acyclic precursor and other stapled 3_10_-helical peptides reported to date.

Kawamoto *et al.* [36] reported the use of the Huisgen 1,3-dipolar cycloaddition reaction to generate single or double triazole-stapled BCL9 α-helical peptides. The interaction between β-catenin and B-cell CLL/lymphoma 9 (BCL9), critical for the transcriptional activity of β-catenin, is mediated by a helical segment from BCL9 and a large binding groove in β-catenin. In their study, they employed the triazole stapling method to stabilize BCL9 α-helical peptides. These peptides increased potency and improved helical structure. The double-stapled peptides (see example **11**, Figure 3) were over 90% helical and demonstrated potent binding to β-catenin. Several of the designed single and double triazole-stapled peptides also showed improved resistance to proteolytic degradation.

### 3.4. Peptide Cyclization

Head-to-tail cyclization is an important approach to increase structural rigidity and stability of peptides. Exoproteases such as aminopeptidases or metallo-carboxypeptidases can recognize N- or C- terminal groups and hydrolyze peptides. As a result, head-to-tail cyclopeptides are relatively more resistant proteolytically than the linear counterparts.

Meldal and coworkers [37] prepared head-to-tail cyclic peptides (**12**, Figure 3) using copper-catalyzed ring closing reactions. They incorporated the azide and alkyne containing amino acids, respectively, in standard Fmoc-based solid phase peptide synthesis. Then two on-resin cyclization methods were applied. In the first method, after removing side-chain protection with trifluoroacetic acid, 2 equiv of CuI and 50 equiv of N,N-diisopropylethylamine were added and kept overnight. Following cleavage from the resin with 1% NaOH, the cyclic peptide was obtained in 76% yield after purification. In the second method, the cyclization was performed in the presence of complete side chain protection and N-terminal Fmoc protection. Under the same reaction condition, the peptide was cyclized and a similar high yield (79%) was obtained. The results confirmed the high selectivity of the reaction, and either protected or non-protected peptide could be cyclized efficiently with click chemistry. Therefore, both methods are successful for the ring-closing reaction and could be used in future studies.

Small-sized head-to-tail peptide ring-closing reactions, for example with tri- or tetra- peptides, are difficult, due to possible oligomerization as well as ring strain prohibiting cyclization [38]. Click chemistry has demonstrated efficacy in tetrapeptide ring closing. Bock *et al*. [38] used the 1,4-disubstituted 1,2,3-triazole as an amide bond surrogate and cyclization aid. A model tetrapeptide, cyclo-[(L)Pro-(L)Val-(L)Pro-(L)Tyr], isolated from bacteria with potential inhibitory activity to tyrosinase, was difficult to synthesize with traditional peptide cyclization. A triazole motif was introduced to help facilitate the ring closure (**5**, Figure 2). They found that the normally difficult-to-form amide bond closure between Pro and Tyr instead succeeded with up to 70% yield using triazole formation. The method demonstrated the potential of click chemistry to derive small cyclic peptide analogues, that are too strained for ring closure via lactamization.

The most frequently reported side reaction for head-to-tail cyclization is dimerization or oligomerization. Solid-phase cyclization has an inherent dilution effect, which favors intramolecular self-cyclization. However, dimers have often been found in products [39,40]. Both solid-phase [39,40,41] and solution-phase cyclization [24,33,34,38,42,43] were investigated intensively to control the intramolecular cyclization. Li has written a thorough review on this topic [44].

## 4. Introduction of Contact Groups to Enhance Affinity

Linking functional groups on peptides using click chemistry provides a convenient and efficient way to increase structural diversity and improve bioactivity.

**Figure 4:**
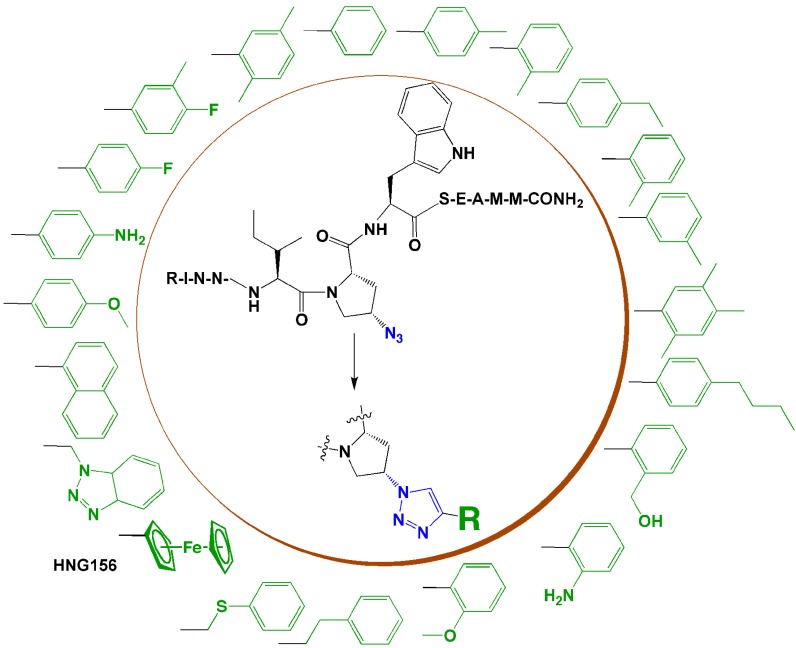
A family of HIV-1 Env antagonist 4-aryl 1,2,3-triazole peptides synthesized *via* click cycloaddition.

**Figure 5:**
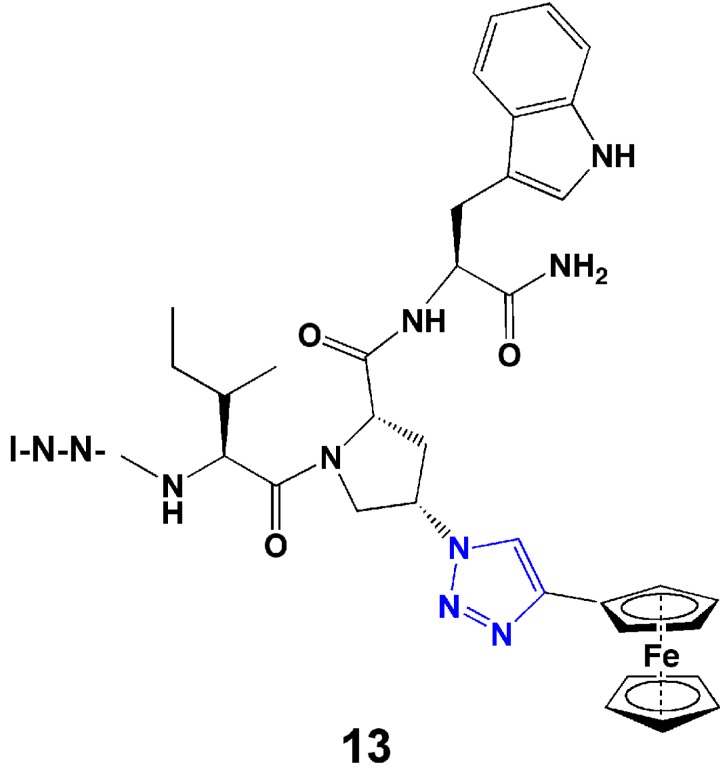
Structure of minimized peptide triazole, UM15.

In the research group of the authors, we developed a series of triazole-incorporated peptide-based inhibitors to target human immunodeficiency virus type-1 (HIV-1) envelope gp120 protein and antagonize cell entry. The parent dodecapeptide, RINNIPWSEAMM, was first discovered by phage display [45]. The Pro residue was chosen for structural modification because of its location next to a Trp residue, which was hypothesized to form a hot spot for stabilizing the interaction with gp120. A *cis*-4-azidoproline substituted for Pro in the parent peptide allowed linkage with a panel of substituted alkynes using CuI catalyzed click chemistry. The substituted alkynes, with diverse aromatic, aliphatic, acidic, or basic features, were used to identify structural characteristics that could provide high binding affinity. Both the peptide syntheses and CuI catalyzed cycloadditions were performed on-resin [46]. A surface plasmon resonance (SPR) optical biosensor was used to characterize the binding affinities of peptides with gp120 [46]. In the initial affinity screen, three aromatic-functionalized alkynes and one cyclohexene-substituted alkyne showed higher affinity than others [46]. Among these four inhibitors, a phenylacetylene-formed phenyl triazole peptide, designated as HNG105, showed a two-order magnitude increase in gp120 binding affinity *vs.* parent dodecapeptide and close-to-nanomolar anti-viral potency. Considering the effect caused by the aromatic substitution, in a follow-up study, a family of 4-aryl 1,2,3-triazole peptides were synthesized (Figure 4) [47,48]. A ferrocenyl substituted peptide triazole HNG156 (Figure 4) was identified that had more than a 700-fold increase in binding affinity (*K*_D_ = 7.4 nM) and 500-fold increase in anti-viral activity (IC_50_ = 96 nM) *vs.* the parent peptide [48]. From this work, a structurally minimized form of peptide triazole has been identified with substantial activity [49] (**13**, Figure 5), leading to the feasibility to design peptidomimetics as drug candidates. The work demonstrates that click chemistry can be an efficient means to increase structural variety and can be used to search for structures that fit a binding target to enhance affinity and bioactivity.

Zhang *et al.* developed a focused dipeptide conjugated azidothymidine (AZT) library for searching substrates of peptide transporter 1 (PEPT 1) [50]. The amino acids, Phe, Val, Leu, Ile, Gly, and Ala, were selected and combinatorially synthesized on the resin. Then, propiolic acid coupling and the [3+2] Huisgen 1,3-dipolar cycloaddition were carried out. Over 60 dipeptide-AZT conjugates containing various dipeptide sequences were screened in a PEPT1 overexpressing cell model for their abilities to compete with the known ligand cephalexin. Several new dipeptide sequences were identified that had a high affinity for PEPT1 and mediated significant activity across intestinal epithelia.

Fragment libraries have been extensively used to screen derivatives important for medicinal chemistry goals [51,52]. Irie *et al.* [53] have successfully identified novel lead compounds for kinase targets using kinase-focused evolved fragment (KFEF) libraries generated using the “click chemistry” methodology. KFEF libraries were prepared using two types of alkyne-azide cycloaddition reactions, a typical Cu-catalyzed click reaction to obtain 1,4-disubstituted 1,2,3-triazoles [4,9] and a recently introduced Ru-catalyzed cycloaddition reaction [54] to construct a 1,5-disubstituted 1,2,3-triazole library. The triazole ring formed in 1,4-disubstituted 1,2,3-triazoles reaction produces planar structures [19], which fit into the narrow ATP binding site of kinases. Screening of this library against a panel of kinases generated several hit compounds having good inhibitory potency. For example, compound “4C4” showed excellent inhibitory effect to kinases FLT3 (IC_50_ = 1.1 μM) and GSK3β (IC_50_ = 0.49 μM), respectively. Copper-click chemistry has been employed [55] for structure–guided rational design of highly potent peptidic inhibitors of the trypsin-like serine protease matriptase based on the monocyclic variant of the sunflower trypsin inhibitor-1 (SFTI-1).

## 5. Bioconjugation to Combine Units with Different Functions

Bioconjugation is a process of covalently linking two or more biomolecular building blocks for the development of bi- and multi-functional molecules. It also encompasses attachment of synthetic molecules to biomolecules such as carbohydrates, peptides, proteins, nucleic acids for various *in vivo* therapeutic and imaging applications. Pioneering work by Sharpless group [9] has enabled click chemistry to evolve as a powerful tool in biomedical research. Click chemistry has gained exceptional importance in bioconjugation because of its bio-orthogonal, biocompatible, chemoselective and stereospecific potential.

### 5.1. Peptide-Carbohydrate Conjugates

**Figure 6:**
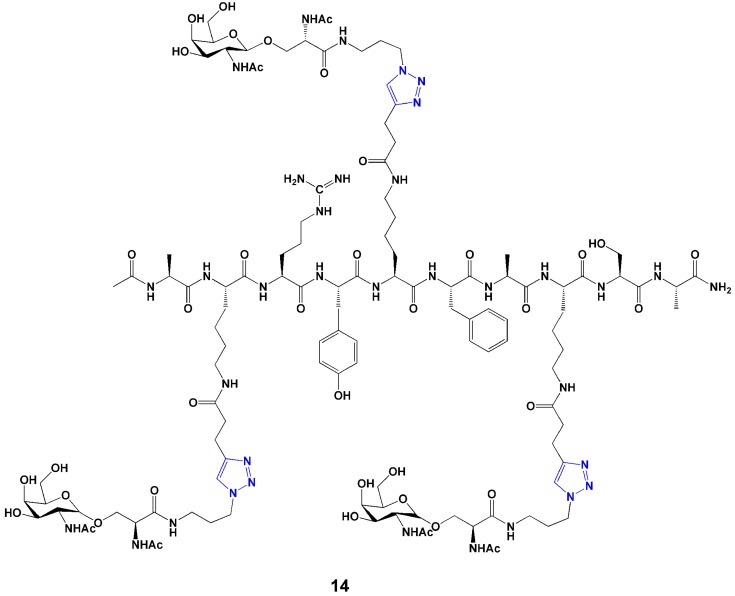
Structure of an anti-cancer carbohydrate-peptide vaccine candidate [56].

Carbohydrates play a major role in different physiological processes from cell signaling to pathogen defense. Carbohydrate-based drugs are still in their infancy because of complexity involved in synthesis and chemical modification. However click chemistry provides a powerful tool to design diverse arrays of protein and peptide-carbohydrate conjugates for a wide range of applications, such as vaccines, therapeutics and antibiotics. A carbohydrate-based anti-cancer vaccine candidate [56], contains several tumor associated oligosaccharide antigens incorporated on a polypeptide backbone through “click-like” cycloaddition between azido-carbohydrate and the alkynyl polypeptide components (**14**, Figure 6).

Glycopeptides occur naturally and display a multitude of biological and pathological functions [57,58]. Extensive research has been pursued in the glycopeptidomimetics field [59,60]. Dondoni and Kuijpers [59,60] reported a range of novel glycopeptide derivatives wherein the bioactive amino acid and carbohydrate moieties were coupled *via* the click reaction. Hybrids containing peptides fused to β-lactam glycoconjugates were designed to bind lectins or carbohydrate recognition domains in selectins. The hybrids contained 1,2,3-triazolylmethyl moiety as the “shape-modulating linker” [61]. Anti-malaria drug candidates have been made involving peptide sugar glycoconjugates. Here, the peptides contained a lysine residue functionalized at the ε-amino group as a propargyloxycarbonyl (alkyne) derivatives. These were selectively conjugated with azide derivatives of carbohydrates or thymidine, using Cu(I) catalyzed click reactions [62]. Such compounds function as potent inhibitor of sir2, NAD^+^ dependent deacetylases of the malaria parasite. Synthesis of anti-freeze neoglycopeptides of biological relevance also exploits the potential of click conjugation [63]. Yang *et al.* [64] examined a series of glycosyl triazolyl acids developed through click reactions as new protein tyrosine phosphatase inhibitors that have potential uses to treat auto-immune disorders.

The peptide–dextran conjugate Dex-(KW)_3_, a polyvalent lytic peptide and chemotherapeutic agent, exhibited significantly enhanced anticancer potency *in vitro* by up to 500-fold compared to monomeric (KW)_3_ without dextran conjugation [65]. Click conjugation of alkynyl dextran with azido peptide has yielded peptides with augmented stability, tumor targeting, and pharmacokinetic properties compared to monomeric peptides [66].

Glycosylated cell-penetrating peptides (CPP’s) have overcome the constraint of non-selective association of the parent compounds. Myristoylation and glycosylation of CPP using click chemistry have enabled control of internalization of bioactive peptides to specific cells and also enhanced their bioavailability [67]. A series of biotinylated cell-permeable integrin inhibitor peptidomimetic derivatives developed by Krissansen and coworkers have been put forth as promising drugs for several inflammatory disorders [68,69].

### 5.2. Conjugation for Drug Delivery

Targeting drug molecules to specific cells and enhancing bioavailability have posed major obstacles to the therapeutic usefulness of peptides as drugs. Click chemistry holds promise for developing efficient drug delivery tools. Considerable research is being done on hydrogels for their ability to encapsulate pharmaceutically active molecules for controlled release. Peptide-based hydrogels conjugated to enzymatically sensitive poly(ethylene glycol) (PEG) have been synthesized by Cu(I)-catalyzed 1,3-dipolar cycloaddition to improve peptide biocompatibility [70]. Hydrogels prepared with Cu(I)-catalyzed 1,3-dipolar cycloaddition reaction between a trypsin- and plasmin-sensitive bis-azido peptide and star-shaped alkyne derivatized PEG moieties have potential use for enzyme-triggered release of drugs at the site of tumors [71]. Parrish *et al.* [72] have adopted an approach of click chemistry for the synthesis of novel aliphatic polyesters, which are more biocompatible and provide sites for further functionalization on the polymer backbone. Novel aliphatic polyesters with acetylene moieties were prepared by controlled ring-opening polymerization and were then grafted with oligopeptides and PEG by Cu(I)-catalyzed click chemistry. This approach enabled the “tailoring” of aliphatic polyesters for biomaterials, tissue engineering and drug delivery, by the incorporation of different types of functionalities such as oligopeptides and PEG substituents [72].

Functionalization of a poly-(amide) based dendrimer has been possible through the use of click conjugation, with the dendrimer able to translocate into the cell more readily than unfunctionalized dendrimer [73]. This was achieved by bioconjugation of membrane-interacting peptide gHA(625–644) (gH625) derived from the herpes simplex virus type 1 (HSV-1) envelope glycoprotein H to the termini of the dendrimer. In this way, the peptidodendrimer scaffold acquired the ability to act as a potential drug-delivery cargo across the cell membrane.

Liposomes have also proven to be important scaffolds to deliver drugs to a biological target. However, there is an emerging need to develop liposomes conjugated with materials to provide improved functionality. Lipid-peptide-PEG conjugates have drawn interest due to their use as triggering molecules in enzymatically sensitive drug delivery systems [74,75,76,77,78]. A case in point is Cu-based click conjugation for the synthesis of a site-specific PEGylated lipopeptide [74]. This study has demonstrated the single-step functionalization of solid-supported lipid bilayers for liposomal drug delivery.

Targeting of anti-microbial peptides (AMPs) can be made more selective and more potent against biological membranes by conjugating to pore forming molecules such as nisin [79]. Peptides have been extensively studied as carriers of diverse cargoes, from small molecule pharmaceuticals to fluorescent dyes, proteins, and nucleic acids, to access different subcellular locations [80,81].Cu-based cycloaddition reactions have been used to deliver an azanitrile-based cysteine protease cathepsin inhibitor to lysosomes [82]. Organelle-specific drug delivery has been investigated using different peptides, such as Tat peptide derived from HIV transactivator protein [83], nucleus localization peptide derived from Simian virus 40 (SV40) T-antigen [84], and N-palmitoylated plasma membrane localization peptide [85].

### 5.3. Radio- and Fluorophore-Labeling

Combined with imaging techniques, peptide labeling has proven to be important to investigate complex biological processes both *in vitro* and *in vivo*. Noninvasive nuclear-imaging techniques, such as positron emission tomography (PET) and single-photon emission computed tomography (SPECT), allow high-resolution quantitative imaging of biochemical processes *in vivo* by detecting the distribution pattern of labeled biomarkers over time. Radiolabeling of biologically active molecules has become an important tool to assess novel drug candidates. ^18^F is particularly attractive, as it can be produced in high yields and has favorable decay characteristics (t_1/2_ =109.8 min). Since the click reaction can be carried out in aqueous media, with mild reaction conditions, high yield and selectivity, it has been applied broadly in the radiolabeling of bioactive peptides. Marik *et al.* [86] first reported the synthesis of ^18^F-labeled peptides through click chemistry. Conjugation of ω-[^18^F]fluoroalkynes to various peptides decorated with 3-azidopropionic acid *via* CuI mediated 1,3-dipolar cycloaddition yielded the desired ^18^F-labeled products in 10 min with yields of 54%–99% and excellent radiochemical purity (81%–99%). Glaser et al used 2-[^18^F]fluoroethylazide reactions with a small peptide library of terminal alkynes in the presence of excess Cu^2+^/ascorbate or copper powder to obtain products with high yield and purity [87]. Among many examples are ^18^F-labeled α_v_β_6_ for *in vivo* imaging of a variety of cell surface receptors [88] and ^18^F-labeled RGD peptides for imaging of tumor integrin α_v_β_3_ [89,90,91]. ^99m^Tc chelate conjugates were developed for SPECT [92,93,94].

Labeling peptides with fluorophores provides a powerful tool to investigate biologically relevant interactions like receptor-ligand-binding, protein structures, and enzyme activities *in vitro* and *in vivo*. Click chemistry has advantages over other labeling techniques due to the inertness of the chemical reporters and of the exogenously delivered probes, as well as the selective and efficient reaction between the reporter and the probe. Baskin *et al.* [15] showed for the first time the use of copper-free conjugation to fluorophore-label a cell surface. The labeling reaction occurred with a comparable kinetics compared to the Cu-catalyzed labeling reaction, and at the same time avoided the potential intrinsic cytotoxicity introduced by copper. Kele *et al.* [95] reported that copper-free and copper-mediated click reactions (both are highly selective and efficient) are viable tools to sequentially conjugate biological targets with two different labels without protecting the functional groups.

### 5.4. Immobilization of Peptides for Separation and Imaging

Click chemistry has also been used to develop peptide-based affinity chromatography reagents. Functionalization of agarose beads with azide and terminal alkyne groups is an important application of click conjugation in producing affinity media for purification [96]. Punna *et al.* [96] prepared an agarose resin conjugated to the C-terminal sequence of HIV protease (QCTLNF), which contains primary amide, carboxylic acid, thiol, and alcohol functional groups. Selective conjugation was facilitated using the click attachment strategy. An extra alkyne-containing amino acid was incorporated at the end of the standard solid-phase peptide synthesis sequence, and the final oligopeptide was cleaved from the resin retaining its N-terminal Fmoc protecting group. The resulting peptide resin has applications in aptamer selection and binding studies [96].

Another striking application of click conjugation was fluorescein functionalization of cowpea mosaic virus. This was achieved by exploiting the functionality on the surface of virus for peptide coupling which decorated the viral surface with azide and alkyne moieties followed by fluorescein conjugation involving thio-ether formation [97]. This application of click chemistry might be useful for various imaging and tracking purposes.

## 6. Conclusions

Click chemistry has been broadly used in peptide-based drug research. Due to structural similarity, it has been used to introduce a surrogate of amide and disulfide bonds. This is very useful for peptide modification to increase the metabolic stability. Due to the selectivity, efficiency, and mild reaction condition of the click reaction, it is a convenient way to link groups together, such as peptide fragments and functional groups, and to achieve peptide cyclization (side-chain to side-chain or head-to-tail). The introduction of triazoles may mimic or promote the secondary structure of the peptide. Further, conjugation with various functional structures or groups, such as carbohydrate, polyethylene glycol, and radiolabeling/photolabeling reagents, can efficiently increase the function of peptides. Though the click chemistry is an appealing method in peptide based drug research, it has been less common so far in developing current clinical drugs [98]. The metabolic pathways of triazoles in biological systems and the safety of copper residue in the human body need further investigation. In addition, the oligomerization side reactions need to be further examined. However, in spite of the potential limitations of click chemistry, the availability of initial peptide drug leads accessed by traditional peptide synthesis makes click chemistry a compelling tool to explore the full potential of peptide building blocks for developing useful drug candidates.
